# Mechanical Thrombectomies Requiring an Increased Number of Passes are Associated with Unfavorable Angiographic Outcomes

**DOI:** 10.1055/s-0046-1818603

**Published:** 2026-04-07

**Authors:** João Paulo Mota Telles, Eduardo Anzolin, Dantas Mageste Ferreira, Pedro Neves Fortunato, Pedro Almeida Martins Pontes, Guilherme Augusto Sousa Alcântara, Michel Eli Frudit

**Affiliations:** 1 Universidade de São Paulo, Faculdade de Medicina, Departamento de Neurologia, São Paulo SP, Brazil.; 2 Universidade de São Paulo, Faculdade de Medicina, Departamento de Radiologia, São Paulo SP, Brazil.

**Keywords:** Stroke, Ischemic Stroke, Thrombectomy, Reperfusion, Endovascular Procedures

## Abstract

**Background:**

Mechanical thrombectomy is the standard treatment for acute ischemic stroke due to large-vessel occlusion, but the impact of multiple retrieval attempts on angiographic success and procedural safety remains uncertain.

**Objective:**

To evaluate the association between the number of thrombectomy passes and the likelihood of unsuccessful reperfusion and procedural complications.

**Methods:**

We analyzed a prospective registry of 291 consecutive patients who underwent mechanical thrombectomy at a tertiary center between 2020 and 2025. The variables collected included clinical, radiological, and procedural data. Unfavorable angiographic outcome was defined as a modified Treatment in Cerebral Ischemia (mTICI) score ≤ 2a. Logistic regressions were used to evaluate the predictors of unfavorable reperfusion and complications.

**Results:**

The mean number of retrieval attempts was of 2.6 ± 1.9. Each additional attempt was associated with a 37% increase in the odds of an unfavorable angiographic outcome (odds ratio [OR] =1.37; 95%CI: 1.18–1.60). Patients requiring ≥ 3 passes presented significantly higher odds of angiographic failure (OR = 2.85; 95%CI: 1.55–5.42) and significantly higher complication rates (OR = 1.98; 95%CI: 1.01–4.03). Complications occurred in 15.2% of the cases, including dissections (5.2%), distal embolization (6.3%), and arterial rupture (2.2%). The rate of favorable mTICI score (≥2b) progressively declined from 76.7% in the first pass to 17.6% for patients with ≥ 6 passes.

**Conclusion:**

Thrombectomy cases requiring passes are strongly associated with a higher risk of angiographic failure and procedural complications. Further studies are necessary to explore the mechanisms behind this association.

## INTRODUCTION

Ischemic stroke is a leading cause of death and disability worldwide. 1 2 Mechanical thrombectomy has become the standard of care for patients with acute ischemic stroke due to large-vessel occlusion, significantly improving outcomes when successful recanalization is achieved. 3 4 5 6 While rapid and complete reperfusion is the primary goal, the procedure often requires multiple passes to retrieve the thrombus. 7 Additional passes may increase the risk of vascular injury, procedural complications, and delays in reperfusion, which have been associated with poorer outcomes. 8

Despite the widespread adoption of thrombectomy, the optimal number of retrieval attempts remains uncertain. Some studies suggest diminishing returns after the first few passes, while others indicate that persistent attempts can lead to successful outcomes without substantially increased risk. 7 8 9 10 Particularly, there seems to be an increasing difficulty in achieving successful recanalization after multiple attempts, although previous literature has reported otherwise. 11

The present article aims to systematically evaluate the association between the number of thrombectomy passes and the likelihood of successful recanalization. By analyzing clinical and procedural data, we seek to identify a threshold beyond which further attempts may be futile or detrimental, thereby informing evidence-based decision-making during endovascular stroke treatment.

## METHODS

We analyzed a prospective register of all patients submitted to mechanical thrombectomy at a university hospital, a tertiary reference center, from 2020 to 2025. The registry includes all procedures performed in this center, without limitations. Mechanical thrombectomy eligibility is evaluated by the neurology team, which decides whether the procedure is indicated. The thrombectomy is performed by experienced interventional neuroradiologists and supervised residents.

The data collected included demographics, score on the (National Institutes of Health Stroke Scale (NIHSS) upon admission, score on the Alberta Stroke Program Early Computed Tomography Score (ASPECTS), time from door to puncture, number of attempts, and final score on the modified Treatment in Cerebral Ischemia (mTICI) score. Successful angiographic outcome was defined as mTICI scores of 2b, 2c, or 3. The NIHSS scores are assessed by two different neurologists, the ASPECTS scores, by two neuroradiologists, and the mTICI scores, by two interventional neuroradiologists.

The following procedural complications were registered: puncture-site hematoma, arterial rupture, dissection, distal embolization to the same territory, embolization to another territory, hypotension, access failure, chest pain, and others.

### Statistical Analysis

The continuous data were expressed as mean ± standard deviation or median and interquartile range (IQR) values, as appropriate according to normality checks using the Shapiro-Wilk's test. The categorical data were expressed as frequencies and percentages. The predictors of unfavorable angiographic outcome were evaluated using logistic regressions. Possible predictors were evaluated in univariate analyses and included in the multivariable analysis if associated with a *p* -value < 0.10. Age was included in the multivariable analysis regardless of statistical significance, due to biological plausibility. The analyses were conducted in the R (R Foundation for Statistical Computing) software.

## RESULTS

A total of 291 patients were included in the final analysis ( Table 1 ). Their mean age was of 63.2 ± 16.1 years, and the mean NIHSS score on admission was of 7.8 ± 1.7. Most occlusions were in the M1 segment of the middle cerebral artery (MCA; n = 182; 62.5%), followed by the internal carotid artery (ICA; n = 73; 25.1%) and the M2 segment of the MCA (n = 21; 7.2%). The median time from door to puncture was 08h44 (IQR = 09h59).

**Table 1:** Patient characteristics (n = 291)

Mean age (years)		63.2 ± 16.1
Mean NIHSS score on admission		17.4 ± 7.2
Mean ASPECTS score		7.8 ± 1.7
Mean time from door to puncture (minutes)		86.8 ± 55.7
Occlusion; n (%)	ICA	73 (25.1)
	M1 segment of the MCA	182 (62.5)
	M2 segment of the MCA	21 (7.2)
	Vertebral	27 (9.3)
	Basilar	6 (2.1)
Collaterals: n (%)	No collaterals	23 (7.9)
	Slow to the periphery	91 (31.3)
	Rapid to the periphery	66 (22.7)
	Slow but complete flow in late venous phase	58 (19.9)
	Complete and rapid flow in entire territory	24 (8.2)

Abbreviations: ASPECTS, Alberta Stroke Program Early Computed Tomography Score; ICA, internal carotid artery; MCA, middle cerebral artery; NIHSS, National Institutes of Health Stroke Scale.

The mean number of retrieval attempts was of 2.6 ± 1.9. Neither age (odds ratio [OR] = 1.01; 95%CI: 0.99–1.03), nor the ASPECTS score (OR = 1.09; 95%CI: 0.91–1.31), nor the NIHSS score on admission (OR = 0.99; 95%CI: 0.96–1.04) was significantly associated with angiographic success ( Table 2 ).

**Table 2:** Influence on favorable mTICI score (≥ 2b)

Univariate analysis	OR (95%CI)
Age	1.01 (0.99–1.03)
ASPECTS score	1.09 (0.91–1.31)
NIHSS score on admission	0.99 (0.96–1.04)
Number of attempts	0.73 (0.62–0.85)
**Multivariable analysis**	
Number of attempts	0.72 (0.61–0.84)
> 1	0.51 (0.25–0.95)
> 2	0.35 (0.18–0.64)
> 3	0.20 (0.11–0.38)
> 4	0.19 (0.09–0.39)
> 5	0.18 (0.04–0.27)
> 6	0.19 (0.05–0.65)

Abbreviations: ASPECTS, Alberta Stroke Program Early Computed Tomography Score; mTICI, modified Treatment in Cerebral Ischemia score; NIHSS, National Institutes of Health Stroke Scale; OR, odds ratio.

**Figure 1:**
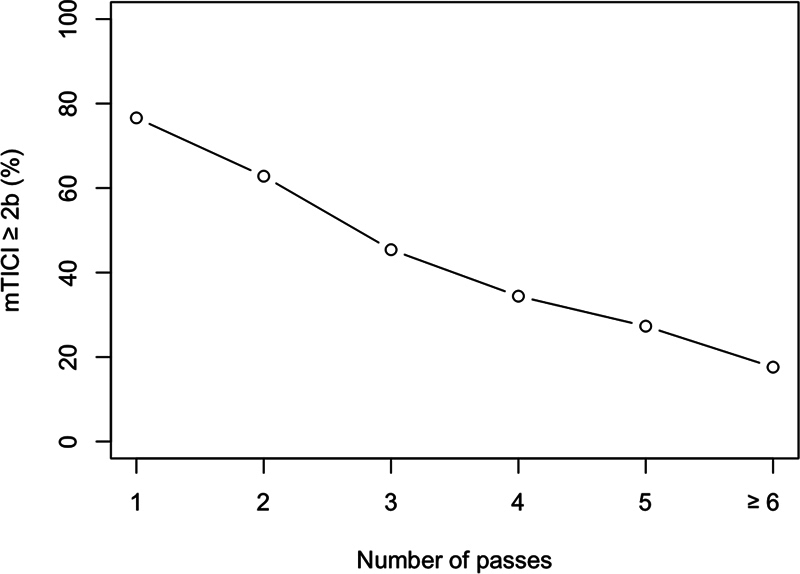
Notes: The graph displays the percentage of favorable angiographic results (mTICI score ≥ 2b) per pass. Overall, 30.9% of the patients (n = 90) achieved successful reperfusion in the first pass. Percentage of favorable angiographic outcome (modified Treatment in Cerebral Ischemia [mTICI] score ≥ 2b) per pass.

Table 3 presents the association between the number of thrombectomy attempts and the likelihood of an unfavorable angiographic outcome, defined as mTICI score ≤ 2a. When modeled as a continuous variable, each additional retrieval attempt was associated with a 37% increase in the odds of an unfavorable outcome (OR = 1.37; 95%CI: 1.18–1.60). Overall, 90 patients (30.9%) achieved reperfusion in the first pass. The rate of favorable mTICI score (≥ 2b) constantly declined from 76.7% in the first pass to 17.6% for patients with ≥ 6 passes ( Table 4 and Figure 1 ).

**Table 3:** Influence of number of attempts on unfavorable mTICI score (≤ 2a)

		Odds ratio (95%CI)	Unfavorable results (%)
Number of attempts*		1.37 (1.18–1.60)	44.8
Each attempt	≥ 2 (n = 174)	2.00 (1.05–3.99)	23.7
	≥ 3 (n = 131)	2.85 (1.55–5.42)	28.5
	≥ 4 (n = 75)	4.85 (2.61–9.14)	40.5
	≥ 5 (n = 42)	5.19 (2.56–10.55)	47.6
	≥ 6 (n = 20)	9.51 (3.67–26.64)	65

Abbreviation: mTICI, modified Treatment in Cerebral Ischemia score.

Notes: Logistic regressions were modeled to evaluate the odds ratio of unfavorable angiographic outcome (mTICI score ≤ 2a) after each attempt. *The number of attempts refers to a regression in which the number of attempts was used as a single continuous variable.

**Table 4:** Favorable angiographic results mTICI (≥ 2b) per number of passes

Number of attempts	mTICI score ≥ 2b: n (%)
1	69 (76.7)
2	27 (62.8)
3	25 (45.4)
4	11 (34.4)
5	6 (27.3)
≥ 6	3 (17.6)

Abbreviation: mTICI, modified Treatment in Cerebral Ischemia score.

Stratified analyses by specific thresholds of attempts further illustrate a dose-response relationship. Compared to patients who required only 1 attempt, those who underwent 2 or more attempts had twice the odds of an unfavorable result (OR = 2.00; 95%CI: 1.05–3.99), with 23.7% of such cases resulting in mTICI score ≤ 2a. The risk progressively increased with the number of passes: patients needing 3 or more attempts had nearly threefold higher odds (OR = 2.85; 95%CI: 1.55–5.42), and those requiring 4 or more had close to fivefold greater odds (OR = 4.85; 95%CI: 2.61–9.14). The odds escalated sharply for 5 (OR = 5.19; 95%CI: 2.56–10.55) and 6 or more attempts (OR = 9.51; 95%CI: 3.67–26.64), with the latter group experiencing unfavorable angiographic outcomes in 65% of the cases.

**Figure 2:**
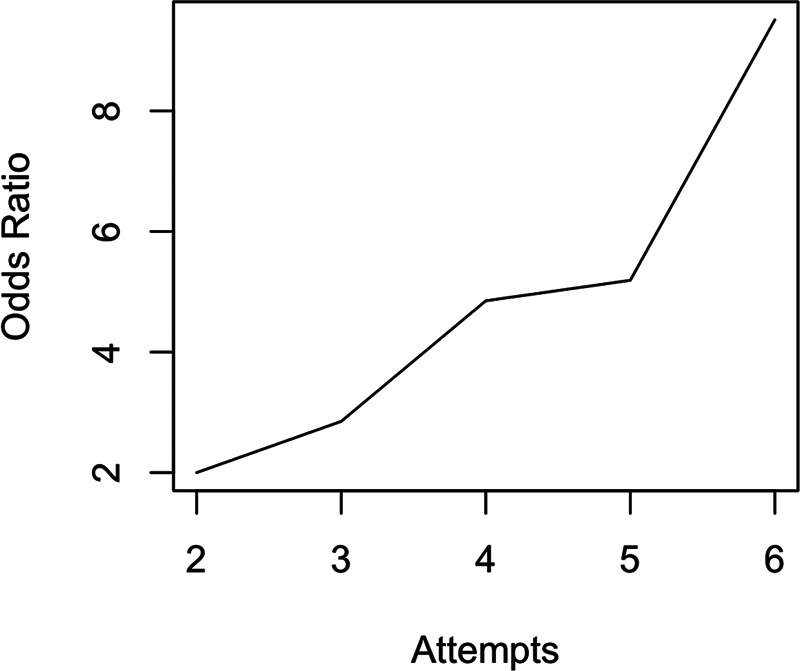
Notes: Logistic regressions were modeled to evaluate the odds ratio of unfavorable angiographic outcome (mTICI score ≤ 2a) after each attempt. A clear and consistent increasing trend is shown, particularly after the third attempt. Number of attempts versus odds ratio of unfavorable angiographic outcome (mTICI score ≤ 2a).

Figure 2 illustrates the stepwise increase in the odds of angiographic failure as the number of attempts increases, with a notable inflection beyond the third attempt. Patients undergoing 5 or more attempts had an OR > 5 for unsuccessful recanalization compared to those with 2 attempts.

There were 41 (15.2%) complications: 2 puncture-site hematomas (0.7%), 6 arterial ruptures (2.2%), 14 cervical artery dissections (5.2%), 17 distal embolizations (6.3%), 2 (0.7%) embolizations to a different territory, and 7 others. The rates of complication were of 12.1% for 1 pass, 9.3% for 2 passes, 21.4% for 3 passes, 18.2% for 4 passes, 13.6% for 5 passes, and 33.3% for ≥ 6 passes. There was a trend toward a higher number of complications with increasing passes (OR = 1.16; 95%CI: 0.98–1.37; *p* = 0.07), with significantly higher odds of complications for patients with ≥ 3 passes (OR = 1.98; 95%CI: 1.01–4.03; *p* = 0.05).

## DISCUSSION

In the current study, we found a strong and progressive association between the number of thrombectomy attempts and the likelihood of an unfavorable angiographic outcome, defined as an mTICI score ≤ 2a. While baseline characteristics such as age, ASPECTS score, and NIHSS score on admission were not significantly associated with recanalization success, the number of passes emerged as an independent predictor of procedural failure. The number of passes was also associated with higher rates of procedural complications.

The stepwise increase in the odds of angiographic failure with each additional attempt—culminating in a nearly tenfold increase in odds for patients requiring six or more passes—suggests a diminishing probability of success with repeated efforts. While the association of multiple passes with inferior functional outcomes is well-established, 9 10 the angiographic improvement yielded by these extra attempts is still controversial. A 2025 meta-analysis 12 has reported somewhat conflicting conclusions that the likelihood of successful recanalization did not change significantly with each pass. However, the results of this article 12 show 40% rates of mTICI score ≥ 2b in the first pass, compared to 21% after 2 passes, and 12% after the third pass. This is consistent with our results, which demonstrate that a higher number of passes does not yield favorable angiographic results.

The underlying mechanisms for this association are likely multifactorial. Repeated device manipulation may contribute to endothelial injury, distal embolization, vasospasm, or clot fragmentation, all of which can compromise reperfusion efficacy and clinical outcomes. 13 14 15 Moreover, the need for multiple passes may reflect more complex clot morphology or challenging vascular anatomy—factors inherently associated with poorer prognosis. 8 Nonetheless, our analysis highlights that even after adjusting for patient- and stroke-related variables, the number of attempts itself remains a significant determinant of procedural success.

While previous studies have explored the relationship between the number of passes and outcomes, their research questions differ from ours. For instance, the ASTER trial 16 only analyzed patients who achieved successful recanalization, assessing whether more than three passes in this subgroup predicted poorer outcomes. In contrast, our analysis includes patients who failed to achieve successful recanalization—representing the real-world dilemma faced by interventionalists deciding whether to attempt another pass when success remains uncertain. Moreover, although Baek et al. 17 addressed a similar question, their results stem from a Korean cohort with substantially-different clinical and system characteristics, including shorter onset-to-recanalization times and higher overall recanalization rates. The present study adds value by providing real-world data from a Brazilian public healthcare setting, reflecting the challenges and outcomes relevant to local practice.

From a practical standpoint, these findings may help inform procedural strategy and decision-making. A meta-analysis 18 has shown that functional outcomes do not differ between patients who achieved mTICI scores of 2b on their first pass compared to 2c or 3 with multiple passes. Connecting these findings with our results, one may argue that further attempts are particularly futile if an intermediate (mTICI score of 2b) result has been achieved.

### Limitations

The current study has several limitations. First, as a retrospective analysis, it is subject to selection bias and residual confounding. Second, our analysis focused on angiographic outcomes, and while clinical endpoints (such as functional independence or mortality) are ultimately most relevant, angiographic success remains a key intermediate determinant of prognosis. Lastly, data on procedural complications were not the primary focus of the present study, and they warrant further dedicated investigation.

In conclusion, the number of thrombectomy attempts is a strong and independent predictor of unfavorable angiographic outcomes. In challenging cases that fail to recanalize after 3 passes, the rates of procedural complications increase significantly, while the angiographic outcomes are significantly worse.
